# Anti-Proliferative and Apoptosis-Inducing Effects of Camptothecin-20(s)-*O*-(2-pyrazolyl-1)acetic Ester in Human Breast Tumor MCF-7 Cells

**DOI:** 10.3390/molecules19044941

**Published:** 2014-04-17

**Authors:** Chu Chu, Jialin Xu, Dongping Cheng, Xingnuo Li, Shengqiang Tong, Jizong Yan, Qingyong Li

**Affiliations:** 1 College of Pharmaceutical Science, Zhejiang University of Technology, Hangzhou 310014, China; E-Mails: chuchu@zjut.edu.cn (C.C.); fantasticxu@sina.cn (J.X.); Chengdp@zjut.edu.cn (D.C.); lixingnuo@zjut.edu.cn (X.L.); sqtong@zjut.edu.cn (S.T.); yjz@zjut.edu.cn (J.Y.); 2 Key Laboratory of Forest Plant Ecology, Ministry of Education, Northeast Forestry University, 332# No. 26 Hexing Road, Harbin 150040, China

**Keywords:** Camptothecin-20(s)-*O*-(2-pyrazolyl-1)acetic ester, MCF-7 cell line, anti-proliferative, apoptosis

## Abstract

Camptothecin-20(s)-*O*-(2-pyrazolyl-1)acetic ester (CPT6) is a novel semi-synthetic analog of camptothecin. In a previous report, CPT6 possessed higher cytotoxic activity *in vitro* towards human breast tumor MCF-7 cells than topotecan. In this study, the antitumor activity of CPT6 on the human breast tumor MCF-7 cell line was analyzed using the MTT method. The underlying mechanism of CPT6 action was investigated by analyzing the cell cycle distribution, apoptotic proportion, changes in mitochondrial membrane potential, and intracellular Ca^2+^ concentration using flow cytometry. Nuclear and mitochondrial morphologies were also observed by laser scanning confocal and transmission electron microscopy. DNA damage was observed in MCF-7 cells treated with CPT6. Low-dose CPT6 had a significant cytotoxic effect and could inhibit proliferation and induce apoptosis in MCF-7 cells, possibly through cell nucleus fragmentation and DNA damage. CPT6 thus appears to display potent antitumor activity against human breast tumor MCF-7 cells via the induction of apoptosis, and may be a useful alternative drug for breast cancer therapy.

## 1. Introduction

**Figure 1:**
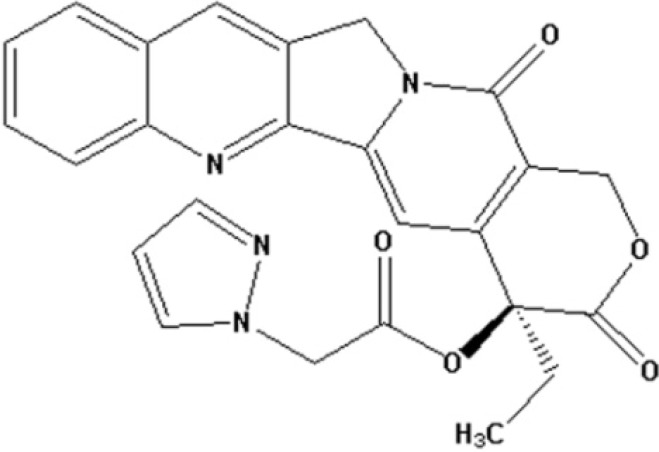
Chemical structure of camptothecin-20(s)-*O*-(2-pyrazolyl-1)acetic ester (CPT6).

Camptothecin (CPT) is an alkaloid originally isolated from *Camptotheca acuminate* by Wall and Wani in 1966 [1], and is one of the most important lead compounds in anticancer research. The antitumor activity of CPT is due to its ability to stabilize the reversible covalent DNA-topoisomerase I complex [2,3]. Topoisomerase I act by breaking and then resealing a single strand of DNA, thus relieving the torsional strain that occurs during DNA transcription and replication. CPT derivatives (CPTs) have been shown to exhibit anticancer activity both *in vitro* and *in vivo*. CPTs represent an important class of anticancer drugs with a wide spectrum of activities in many solid tumors, including lymphoma, gastric cancer and colorectal cancer. Further evidence indicates that the lipophilic CPTs show improved cytotoxicity and inhibition of topoisomerase I, compared with the water-soluble CPTs [4,5]. In previous studies, we introduced nitrogenous heterocyclic aromatic groups in the 10- and 20-positions of CPT and obtained a series of CPT analogs, some pyridine quaternary salt derivatives of which showed good water solubility and potent antitumor activity both *in vitro* and *in vivo* [6,7]. Other nitrogenous heterocyclic aromatic groups introduced in the 10- or 20-postion can enhance the stability of the lactone component and the antitumor activity [6,7]. Derivatives modified by pyrazole in the 10- or 20-position all showed notable antitumor activity *in vivo* and stability of the lactone component *in vitro*. The antitumor activity was not only related to the substituted positions, but also to the substituent. Among these derivatives, camptothecin-20(s)-*O*-(2-pyrazolyl-1)acetic ester (CPT6, Figure 1) exhibited good antitumor activity *in vitro*, and demonstrated stronger cytotoxicity towards the human breast tumor MCF-7 cell line. The antiproliferative activity and the mechanism of action of CPT6 against human breast tumor MCF-7 cells, however, have not been reported. In this study, the antiproliferative activity of CPT6 against human breast tumor MCF-7 cells was analyzed using the MTT method. The mechanism of CPT6 action against human breast tumor MCF-7 cells was also investigated by flow cytometry, laser scanning confocal microscopy (LSCM) and transmission electron microscopy (TEM).

## 2. Results and Discussion

### 2.1. Antiproliferative Activity of CPT6

**Figure 2:**
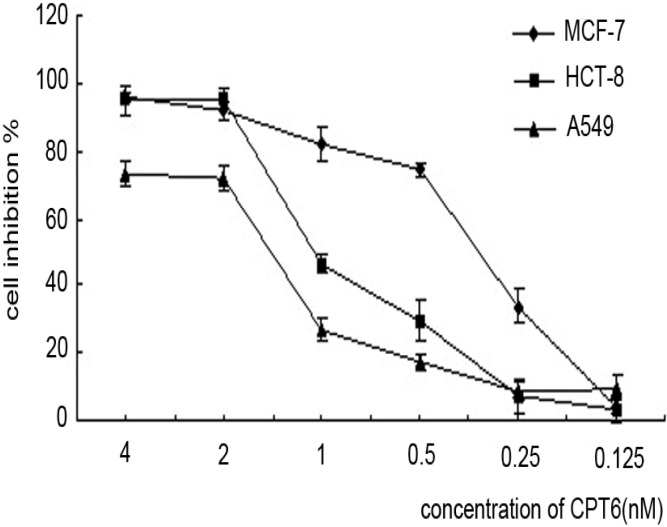
The inhibitory effects of three cell lines treated with CPT6 for 72 h.The experiments were performed in triplicate. Data are presented as mean ± SD of three independent experiments.

The antiproliferative activity of CPT6 was determined in three cancer cell lines: human breast cancer MCF-7 cells, human colon cancer HCT-8 cells, and non-small cell lung cancer A549 cells. As shown in Figure 2, MCF-7 cells were the most sensitive of the three cell lines to CPT6 within 72h. The IC_50_ value of CPT6 against MCF-7 cell was 0.46 ± 0.02 nM, which was more effective than CPT (IC_50_ = 0.65 ± 0.04 nM) and TPT (IC_50_ = 1.64 ± 0.05 nM). The growth of MCF-7 cells was significantly inhibited in a dose and time-dependent manner in the presence of CPT6 (0, 0.125, 0.25, 0.5, 1, 2, 4 nM) at 24, 48, and 72 h (Table 1).

**Table 1:** Cells were incubated with CPT6 (from 0.125 to 4 nM) for 24 h, 48 h and 72 h (* *p* < 0.05, ** *p* < 0.01).

CPT6 (nM/mL)	Inhibition (%) ± SD		
	24 h	48 h	72 h
0.125	8.55 ± 2.68 *	6.96 *±* 2.44 *	10.77 *±* 1.48 **
0.25	14.09 *±* 3.05	18.02 *±* 3.11	32.88 *±* 3.07
0.5	27.10 ± 2.69 *	30.82 *±* 3.59	52.83 *±* 4.01
1	36.69 *±* 3.29	43.56 *±* 2.59 *	64.31 *±* 3.13
2	48.53 *±* 3.62	53.73 *±* 3.15	73.94 *±* 3.82
4	56.80 *±* 2.42 *	67.36 *±* 3.22	80.63 *±* 4.16

### 2.2. Analysis of Cell Cycle Distribution

**Figure 3:**
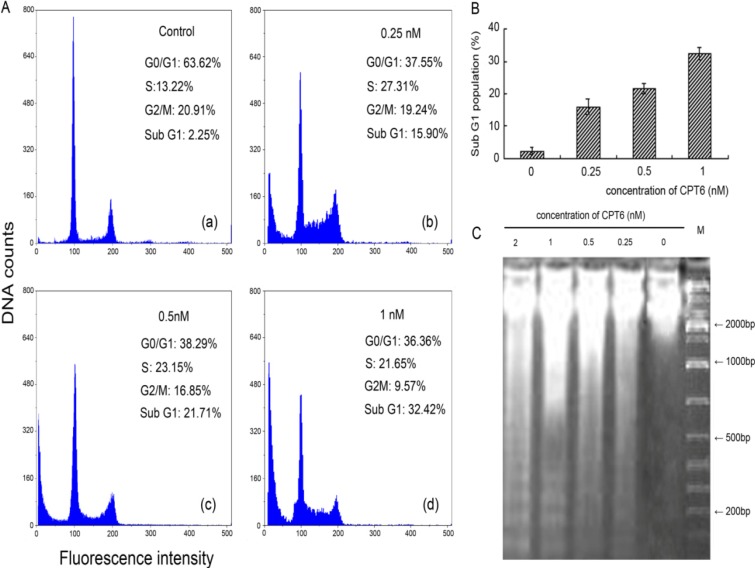
CPT6 induces apoptosis in MCF-7 cells at 48 h. **A**: Flow cytometric analysis of MCF-7 cell cycle distribution (excitation wavelength: 351 nm; emission wavelength: 380 nm). (**a**) Control group; (**b**) treatment with 0.25 nM CPT6; (**c**) treatment with 0.5 nM CPT6; (**d**) treatment with 1 nM CPT6. **B**: The proportional (%) of Sub-G1 phase population. **C**: DNA fragmentation of apoptotic MCF-7 cells treated with CPT6 at 0, 0.25, 0.5, 1 and 2 nM for 48 h.

The cell cycle distribution was analyzed using Multicycle software (Partec GmbH, Münster, Germany). A significant increase in the percentage of cells in Sub-G1 phase was found after treatment with CPT6, compared with control cells (Figure 3). MCF-7 cells was significantly inhibited in a dose-dependent manner in the presence of CPT6. As shown in Figure 3B, when cells were exposed to CPT6 (1 nM) for 48 h, the percentage of Sub-G1population showed a marked increase (32.42%) compared with the control (2.25%). G0/G1 phase were decrease along with the increase of Sub-G1 phase, and the population of cells treated in S phase increased significantly compared with that of control cells. All these data indicate that CPT6 affects the replication of DNA and cause cell cycle arrest in S phase, prevent the cell proliferation. The cell proliferation inhibition causes increased cells entering in Sub-G1 phase, subsequently induces cell apoptosis. So we can deduce that CPT6 may induce apoptosis by causing DNA fragmentation.

### 2.3. DNA Damage

MCF-7 cells were treated with CPT6 (0, 0.25, 0.5, 1, 2 nM) for 48 h and apoptosis was confirmed using the DNA laddering method. Obvious diffusion bands of DNA fragments representing multiples of the internucleosomal DNA length (about 180–200 bp) were detected (Figure 3C). The apparent densities of the DNA fragment bands were increased in the treated group, compared with untreated cells, along with increasing concentrations of CPT6. Control cells did not exhibit any such DNA fragmentation. Such a pattern corresponds to inter-nucleosomal cleavage, which is characteristic of apoptosis.

### 2.4. Apoptosis/Necrosis Analysis

**Figure 4:**
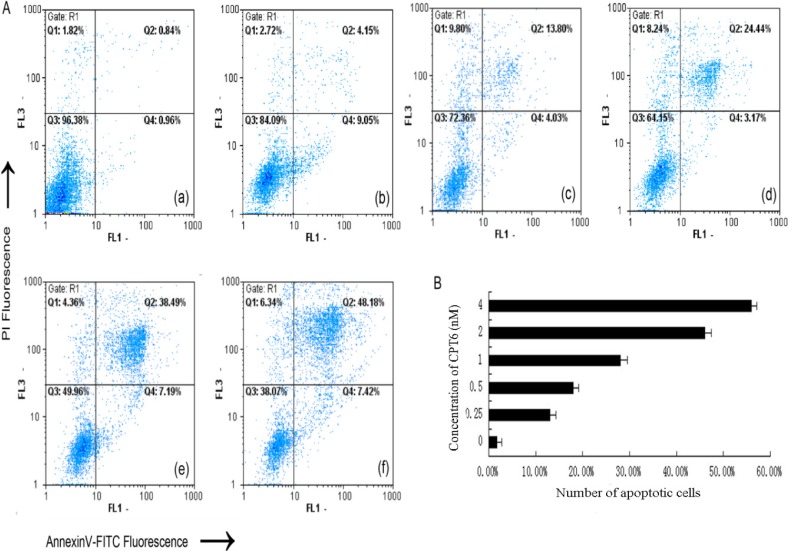
Flow cytometric analysis of CPT6-induced apoptosis in MCF-7 cells for 48h using the annexin V-fluorescein isothiocyanate/propidium iodide method. **A**: (**a**) Control group; (**b**) treatment with 0.25 nM CPT6; (**c**) treatment with 0.5 nM CPT6; (**d**) treatment with 1 nM CPT6; (**e**) treatment with 2 nM CPT6; (**f**) treatment with 4 nM CPT6. **B**: the apoptotic cells (%) stained with annexin V positive according to every concentration of CPT6. The data represent the means of three independent experiments and the corresponding standard deviation.

To elucidate the mode that CPT6 used to exert cytotoxicity, apoptosis or necrosis, experiments were conducted by using MCF-7 cells. The disruption of cell membrane phospholipid asymmetry, evidenced by phosphatidylserine (PS) externalization, was examined by utilizing annexin V-FITC and propidium iodide assay and monitored via flow cytometer protocol. The cell distributions in flow cytometric histograms are as follow: cells in the lower left quadrant (Q3) represented live cells, negative for annexin V and PI; the lower right quadrant (Q4) represented early apoptotic cells; the upper right quadrant (Q2) represented late apoptotic cells; and upper left quadrant (Q1) represent necrotic cells (Figure 4A). The addition of both early and late apoptotic cells (annexin V-FITC positives) was defined as the total percentage value of apoptotic cells. When MCF-7 cells were treated with CPT6 (0, 0.25, 0.5, 1, 2, 4 nM) for 48 h, the percentage of apoptotic cells increased from 1.80% to 55.60% (Figure 4B). This indicates that CPT6 was able to induce apoptosis of MCF-7 cells in a dose dependent manner.

### 2.5. Changes in Nuclear Morphology and Chromatin Condensation

**Figure 5:**
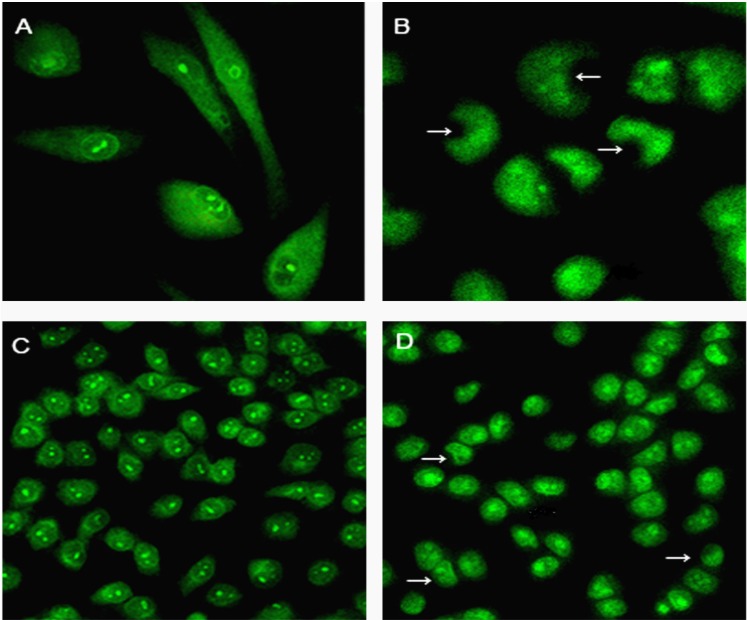
Morphological changes in MCF-7 cells treated with CPT6 for 48 h examined by laser scanning confocal microscopy(excitation: 488 nm, emission: 535 nm). Cultured cells were stained with acridine orange. Bars indicate lunate morphology of chromatin and condensed chromatin. (**A**) Control group; (**B**) treatment with 0.5 nM CPT6 (cells were observed at ×60 magnification); (**C**) control group; (**D**) treatment with 0.5 nM CPT6 (cells were observed at ×20 magnification ).

CPT6 treatment resulted in many visible morphological changes in the nuclear chromatin of MCF-7 cells. In untreated cells, the cell membrane was orbicular and the nuclei were sharp and regular (Figure 5A, C). MCF-7 cells treated with 0.5 nM CPT6 for 48 h showed lunate morphology of chromatin and condensed chromatin (Figure 5B). Moreover, the membranes of the treated cells were shrunken and the nuclei were crescent- or even strip-shaped, which is a typical characteristic of apoptotic cells (Figure 5D).

### 2.6. Morphological Observation of Cell Ultrastructure by TEM

**Figure 6:**
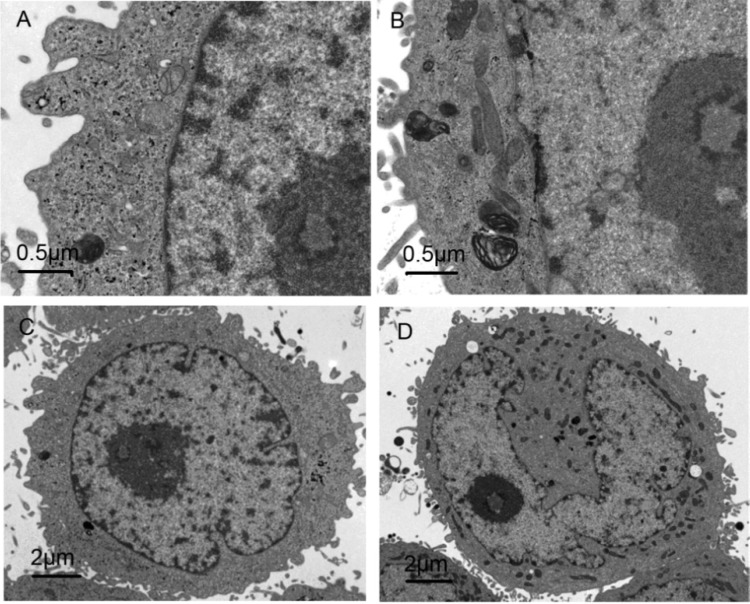
Assessment of cell ultrastructures treated with CPT6 for 48 h using transmission electron microscopy. (**A**) Control group; (**B**) treatment with 0.5 nM CPT6 (cells were observed at ×5000 magnification); (**C**) control group; (**D**) treatment with 0.5 nM CPT6 (cells were observed at ×16,500 magnification).

Untreated cells showed typical ultrastructure characterized by rounded nuclear membranes, regular mitochondria and nuclei containing a nucleolus and euchromatin (Figure 6A, C). However, in treated cells, the nuclear membrane became unclear, the chromatin condensed and the mitochondria were damaged, and more myeloid body could be seen outside the nuclear membrane (Figure 6B). Vacuoles appeared in the cytoplasm and the nuclear membrane was umbilicate (Figure 6D). These early signs of apoptosis were not visible in untreated cells. These observations indicate that 0.5 nM CPT6 induced early apoptosis in MCF-7 cells treated for 48 h.

### 2.7. CPT6-Induced Loss of ΔΨ m in MCF-7 Cells

**Figure 7:**
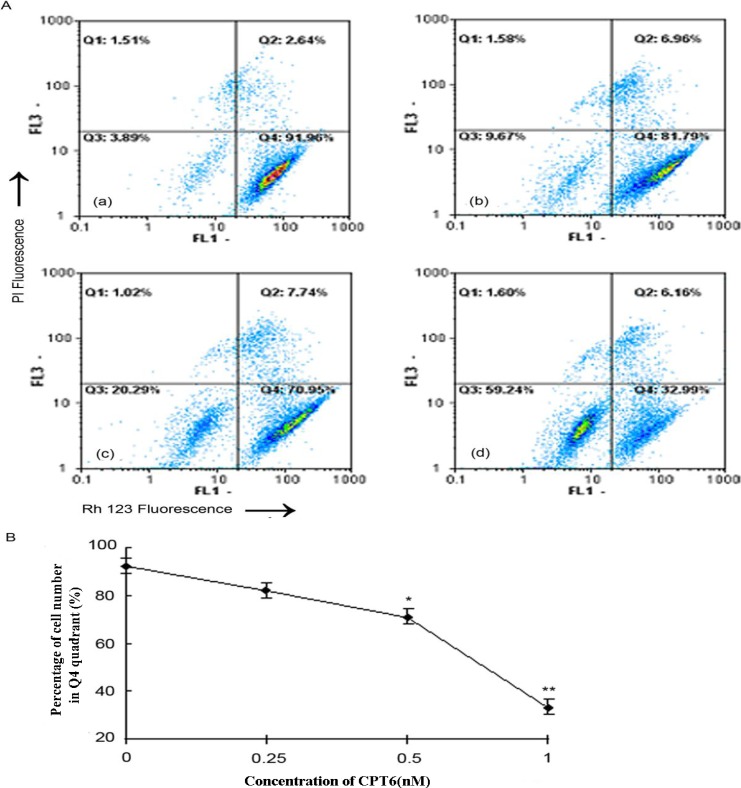
Flow cytometric analysis of the changes in the mitochondrial membrane potential (Δ*Ψ* m) in MCF-7 cells treated with CPT6 for 48 h. **A**: (**a**) Control group; (**b**) treatment with 0.25 nM CPT6; (**c**) treatment with 0.5 nM CPT6; (**d**) treatment with 1 nM CPT6. **B**: Reduction in Δ*Ψ* m in MCF-7 cells treated with CPT6. The data was obtained according to the percentage of cell number in Q4 quadrant which elicited strong fluorescence, resulting from three independent experiments (* *p* < 0.05, ** *p* < 0.01); *p* value compared with the control group (0 nM CPT6).

To evaluate the role of mitochondria in CPT6-induced apoptosis, we investigated the effects of CPT6 on Δ*Ψ* m. Rh123 is available to stain the mitochondria of live cells, and elicit fluorescence. When mitochondria are damaged and Δ*Ψ* m decreases, fluorescence from Rh123 will reduce. So Δ*Ψ* m can be measured by the number of the cells with strong fluorescence. Untreated MCF-7 cells elicited maximal Rh123 fluorescence, reflecting intact functional mitochondria. CPT6 treatment resulted in a rapid dose-dependent dissipation of Δ*Ψ* m, detected by a consequent decrease in mean fluorescence (Figure 7A, B). Δ*Ψ* m was reduced by 59.24% when the cells were treated with 1nM CPT6. These data showed that CPT6 could induce apoptosis in MCF-7 cells, accompanied by alterations in Δ*Ψ* m.

### 2.8. Effect of CPT6 on Intracellular Calcium in MCF-7 Cells

**Figure 8:**
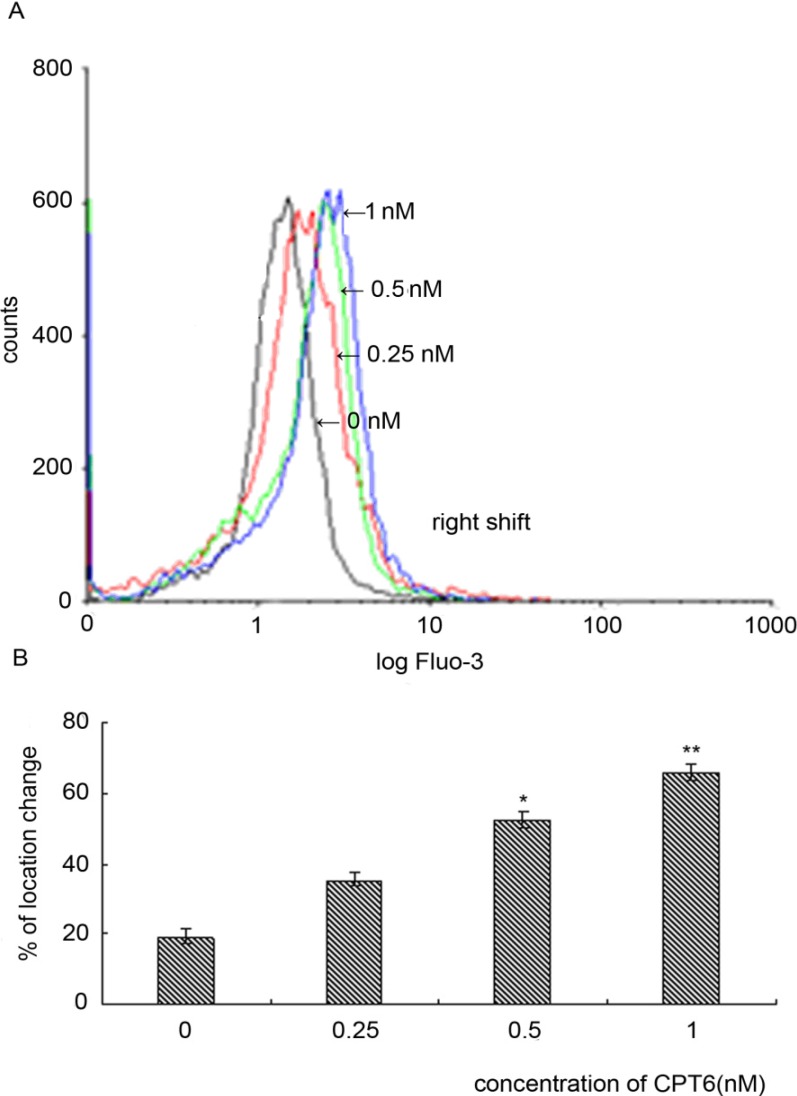
Changes in intracellular calcium in MCF-7 cells treated with CPT6 for 48h. **A**: Black line represents control group; red line represents cells treated with 0.25 nM CPT6; green line represents cells treated with 0.5 nM CPT6; blue line represents cells treated with 1 nM CPT6 (excitation: 488 nm; emission: 525 nm). **B**: Change in calcium levels in MCF-7 cells. The results were reproducible in three additional independent experiments (* *p* < 0.05, ** *p* < 0.01); *p* value compared with the control group (0 nM).

To investigate the role of Ca^2+^ signaling in CPT6-induced apoptosis of MCF-7 cells, we determined the intracellular Ca^2+^ concentration using flow cytometry. The fluorescence intensities of the control and treated groups were not coincident (Figure 8A), and the intensity of the treated group was shifted to the right [8]. WinMDT 2.9 software (Partec GmbH) was used to overlay the absorption peaks and the values of the peak locations were then calculated using CellQuest software. The intracellular Ca^2+^ increased from 19.03% *±* 2.12% to 64.52% *±* 2.48%. The histogram indicated that CPT6 could significantly enhance the intracellular Ca^2+^ concentration in MCF-7 cells (Figure 8B) (* *p* < 0.05, ** *p* < 0.01).

## 3. Experimental

### 3.1. Materials

Dulbecco’s modified Eagle’s medium (DMEM) and RPMI-1640 medium were purchased from HyClone (Logan, UT, USA). Fetal bovine serum (FBS), penicillin, streptomycin and trypsinase were purchased from Tianjin HaoYang Biological Manufacture Co., Ltd. (Tianjin, China). 3-(4,5-Dimethylthiazol-2-yl)-2,5-diphenyltetrazolium bromide (MTT), acridine orange (AO), dimethyl sulfoxide (DMSO), rhodamine 123 (Rh123) and propidium iodide (PI) were purchased from Sigma Chemical Co. (St. Louis, MO, USA). Phosphate-buffered saline (PBS, pH 7.2–7.6) was purchased from Wuhan Boster Biological Technology Ltd. (Wuhan, China). CPT(98%) and TPT(98%) was purchased from Zhejiang Yixin Pharmaceutical Co., Ltd. (Zhejiang, China). For *in vitro* experiments, CPT6 was synthesized as in our previous studies [7]. Its purity was 98.8%, as determined by high-performance liquid chromatography. CPT6, CPT and TPT were dissolved in 300 μL DMSO at a concentration of 6 μM as stock solutions. Dilutions of the compounds were made up in 2% culture medium before each experiment. The final concentration of DMSO did not exceed 0.1% (v/v) and was nontoxic to the cells.

### 3.2. Cell Line and Culture

The human breast cancer cell line MCF-7, the colon cancer cell line HCT-8, and the non-small cell lung cancer cell line A549 were purchased from the Cell Bank of the Institute of Materia Medica, Chinese Academy of Medical Sciences. All cells were grown in DMEM or a nutrient medium (RPMI-1640) supplemented with 10% FBS, 100 μg/mL of streptomycin and 100 U/mL of penicillin, in a water-saturated atmosphere of 5% CO_2_ at 37 °C.

### 3.3. Cytotoxicity and Antiproliferative Activities

Cell proliferation was measured using the MTT assay. Cells in the exponential growth phase were seeded in a 96-well plate (1 × 10^6^ cells/well) and routinely cultured for 24 h. CPT6, CPT and TPT was added at serial concentrations (from 0.125 to 4 nM). 2% culture medium (with 0.1% DMSO) alone was used as a control. The cells were then incubated for 24, 48 and 72 h. MTT PBS solution (20 μL of 5 mg/mL) was then added to 100 μL and incubated for 4 h at 37 °C. The supernatant was removed and the formazans were dissolved in 100 μL DMSO per well. After 10 min shaking, the absorbance was read at 492 nm (with 630 nm as a reference) on a microplate reader (Bio-Tek, America). Assays were performed in three independent experiments.

### 3.4. Cell Cycle Distribution

To analyse the cell cycle phase distribution, MCF-7 cells (1 × 10^6^ cells/well) were seeded in a 6-well plate for 24 h and then exposed to CPT6 (0, 0.25, 0.5, 1 nM). After incubation for 48 h, the untreated cells and treated cells were washed twice with precooled PBS, and then fixed in 70% ethanol at 4 °C [9]. Fixed cells were stained with 200 μL 4'-6-diamidino-2-phenylindole (DAPI) for 10 min in the dark. The cell cycle distribution of 10,000 cells was determined by flow cytometry (Partec GmbH,) using the FloMax software (Partec GmbH).

### 3.5. Detection of DNA Fragmentation

DNA fragmentation assay was used to identify apoptosis. Briefly, 5 × 10^5^ cells were harvested and treated with CPT6 (0, 0.25, 0.5, 1, 2 nM) for 48 h. The supernatant and adherent cells were then collected in a centrifuge tube, washed twice with cold PBS and resuspended in 200 μL PBS. Genomic DNA was isolated using an Apoptosis DNA Ladder Detection kit (Nanjing KeyGEN Biotech. Co. Ltd., Nanjing, China). DNA samples were separated by electrophoresis in a 2% agarose gel for 150 min and visualized by ethidium bromide staining.

### 3.6. Apoptosis Assays

Apoptosis assay was performed using rh Annexin V/FITC Kit (Bender MedSystems GmbH, Wien, Austria), and the fluorescence intensities of FITC-conjugated annexin-V and PI in cells were analyzed using flow cytometry [10,11]. MCF-7 cells were cultured in medium containing different concentrations of CPT6 (0,0.25, 0.5, 1, 2, 4 nM) for 48 h, collected and washed twice with PBS, gently resuspended in 195 μL annexinV-FITC binding buffer (1×) and incubated with 5 μL annexinV-FITC in the dark for 10 min at 25 °C. The cells were then centrifuged at 3000 rpm for 5 min, gently resuspended in 190 μL annexinV-FITC binding buffer (1×) and 10 μL PI was added in an ice bath, followed by immediate analysis by flow cytometry CellQuest software (Partec GmbH).

### 3.7. Morphological Observations

MCF-7 cells were grown on glass coverslips at a density of 2 × 10^4^ cells/well in 6-well plates. After adhering for 24 h, the cells were washed twice with cold PBS and treated with or without 0.5 nM CPT6 for 48 h at 37 °C. The cells were then washed twice with cold PBS and stained with AO (20 μg/mL) for 5 min at 37 °C, then immediately viewed by LSCM (Nikon ECLIPSE TE2000-E, Tokyo, Japan) at an excitation wavelength of 488 nm and an emission wavelength of 535 nm [12,13].

Ultrastructural analysis was performed by TEM (Tecnai G^2^, Holland). Cells were treated with or without 0.5 nM CPT6 for 48 h, washed with PBS, collected, and pre-fixed in 2.5% glutaraldehyde in 0.1 M phosphate buffer at 4 °C for 2 h. Cells were then washed three times with distilled water and post-fixed in 1% OsO_4_ at 4 °C for 1 h. After fixation, cell blocks were dehydrated through graded concentrations of acetone and embedded in Epon812 (SPI Supplies, West Chester, PA, USA). Cell ultrastructure was analyzed in ultrathin sections by TEM after staining with uranyl acetate and lead citrate [14,15].

### 3.8. Assessment of Mitochondrial Membrane Potential (ΔΨ m)

*ΔΨ m* was measured by flow cytometry with Rh123 and PI double staining [16]. Rh123 and PI (10 mg/L) were dissolved in DMSO and diluted in PBS before use. MCF-7 cells (1 × 10^6^ cells/well) were seeded in a 6-well plate. After adherence for 24 h, the cells were cultured in medium containing different concentrations of CPT6 (0, 0.25, 0.5, 1 nM) for 48 h. Cells were then washed twice with PBS and finally harvested in chilled PBS containing Rh123. The samples were incubated at 37 °C for 30 min in the dark. The cells were collected at 3000 rpm for 5 min, resuspended in PBS and 10 μL of PI was added for 10 min in the dark, followed by immediate analysis by flow cytometry.

### 3.9. Determination of the Concentration of Calcium in Cells

MCF-7 cells (1 × 10^6^ cells/well) were seeded in a 6-well plate. After adherence for 24 h, the cells were washed with cold PBS and treated with different concentrations of CPT6 (0, 0.25, 0.5, 1 nM) for 48 h. Fluo-3/AM (Sigma, 5 μM) was added to the treated cells for 30 min at 37 °C. The cells were then analyzed immediately by flow cytometry using WinMDT 2.9 software (Partec GmbH) and Cell Quest software (Partec GmbH, Münster, Germany).

### 3.10. Statistical Analysis

Results were expressed as mean ± SD. Statistical analysis was performed using Student’s*t-*test. Significance was established at *p* < 0.05.

## 4. Conclusions

Structure-activity studies of CPTs have revealed that positions 7, 9 and 10 of CPT can be modified with no loss of activity [17]. In our previous studies, CPTs modified in the 10- or 20-position by pyrazole all showed notable antitumor activity *in vivo*, and stability of the lactone component *in vitro*. Further, we evaluated that the growth inhibitory activity of CPT6 was associated with the induction of DNA damage and apoptosis in MCF-7 cells.

Due to the critical role of apoptosis in cellular homeostasis, compounds able to induce apoptosis are considered to be potential antitumor agents for cancer treatment [18,19]. Apoptosis is a physiological process that functions as an essential mechanism of tissue homeostasis and is regarded as the preferred way to eliminate unwanted cells [20]. CPT and its derivatives are able to form cleavable drug-enzyme-DNA complexes that inhibit the DNA relegation step [21]. Collision between the DNA replication fork and these complexes has been proposed as a means of explaining the CPT-driven S phase-specific cytotoxicity and the arrest of cells in the G2-M phase of the cell cycle [22]. Apoptosis is related to cell cycle arrest, which is a complicated process that can be regulated by numerous distinct growth signals [23,24]. In the present study, MCF-7 cells treated with CPT6 revealed DNA fragmentation with appearance of the Sub G0/G1 peak indicative of apoptosis, and showed a dose-dependent generation of apoptosis-specific Sub-G1 changes and apoptotic rates. This could be explained by the ability of the CPT6 to induce apoptosis and thus effectively inhibit DNA unwinding and replication [25].

Annexin-V binding is based on the transposition of phosphatidylserine from the inner to the outer face of the cell membrane during the early stages of apoptosis. With fluorescent probe FITC to mark Annexin-V, it directly detected phosphatidylserine valgus which was an important feature of cell apoptosis [26]. CPT6-treated cells clearly showed apoptotic morphologies and the apoptotic rate of the treated cells increased in a dose-dependent manner. The formation of apoptotic bodies, condensed chromatin in MCF-7 cells treated with CPT6 were indicative of apoptosis. CPT6 deformed the cancer cell membranes and caused shrinkage and bleb formation, which are also important morphological characteristics of apoptosis. Disruption of Δ*Ψ* m is one of the earliest intracellular events that occurs following the induction of apoptosis [27,28]. The decrease in Δ*Ψ* m in MCF-7 cells treated with CPT6 was dose-dependent, suggesting that the Δ*Ψ* m may be one significant appearance in MCF-7 cell apoptosis by CPT6. Ca^2+^ is an important signal transducer, not only for intracellular communication but also for intercellular communication. Many studies have demonstrated that an increase in intracellular Ca^2+^ plays a primary role in triggering the apoptosis pathway [29]. MCF-7 cells treated with CPT6 showed a distinct, dose-dependent increase in intracellular Ca^2+^.

At the same time, both DNA fragmentation and flow cytometry analyses showed dose-dependent increases in representative apoptotic characteristics, such as DNA fragmentation and an increase in cells in Sub-G1 phase, the apoptotic cells were increased along with the concentration of CPT6. These results suggest that higher concentrations of CPT6 induced irreparable DNA damage and apoptosis in MCF-7 cells.

In summary, this study provides the first results regarding the antiproliferative and apoptosis-inducing effects of CPT6. CPT6 had a significant cytotoxic effect and was able to inhibit proliferation and induce apoptosis in MCF-7 cells at low doses. CPT6 displayed greater antitumor activities in MCF-7 cells, compared with TPT and CPT at 72h. Although many details of the effects of CPT6 remain to be elucidated, the *in vitro* findings of the present study provide the basis for future *in vitro* and *in vivo* studies and clinical applications.
